# Antarctic ice mass variations from 1979 to 2017 driven by anomalous precipitation accumulation

**DOI:** 10.1038/s41598-020-77403-5

**Published:** 2020-11-23

**Authors:** Byeong-Hoon Kim, Ki-Weon Seo, Jooyoung Eom, Jianli Chen, Clark R. Wilson

**Affiliations:** 1 Division of Glacial Environment Research, Korea Polar Research Institute, Incheon, 21190 Republic of Korea; 2 Department of Earth Science Education, Seoul National University, Seoul, 08826 Republic of Korea; 3 Department of Earth Science Education, Kyungpook National University, Daegu, 41556 Republic of Korea; 4 Center for Space Research, University of Texas at Austin, Austin, TX 78759 USA; 5 Department of Geological Sciences, Jackson School of Geosciences, University of Texas at Austin, Austin, TX 78712 USA

**Keywords:** Climate sciences, Solid Earth sciences

## Abstract

Antarctic ice mass balance is determined by precipitation and ice discharge, and understanding their relative contributions to contemporary Antarctic ice mass change is important to project future ice mass loss and resulting sea level rise. There has been evidence that anomalous precipitation affects Antarctic ice mass loss estimates, and thus the precipitation contribution should be understood and considered in future projections. In this study, we revisit changes in Antarctic ice mass over recent decades and examine precipitation contributions over this period. We show that accumulated (time-integrated) precipitation explains most inter-annual anomalies of Antarctic ice mass change during the GRACE period (2003–2017). From 1979 to 2017, accumulated Antarctic precipitation contributes to significant ice mass loss acceleration in the Pacific sector and deceleration in the Atlantic-Indian Sectors, forming a bi-polar spatial pattern. Principal component analysis reveals that such a bi-polar pattern is likely modulated by the Southern Annular Mode (SAM). We also find that recent ice mass loss acceleration in 2007 is related to a variation in precipitation accumulation. Overall ice discharge has accelerated at a steady rate since 1992, but has not seen a recent abrupt increase.

## Introduction

A rise in global mean sea level (GMSL) is generally associated with global warming, and there is great interest in projecting future changes in GMSL in climate forecasts. The rate of GMSL increase is estimated at about 1.9 mm/year during the twentieth century^1^, increasing to 3.1 mm/year during the past three decades^2^. The increase in GMSL rate in recent decades is mostly attributed to water mass inflow from mountain glaciers (0.7 mm/year) and from the Greenland Ice Sheet (GrIS) (0.5 mm/year)^2^. During the same period, the Antarctic Ice Sheet (AIS) has contributed about 0.3 mm/year to GMSL, roughly half the amount from the GrIS. However, the Ice sheet Mass Balance Inter-comparison Exercise 2 (IMBIE2)^3^ reported recently that the rate of AIS ice mass loss has evidently increased during the last decade; the rate was estimated to about − 47 Gton/year during 1992–2006, increasing to − 194 Gton/year during 2007–2017. If such an acceleration of AIS ice mass loss continues, then the AIS would soon become a larger contributor to GMSL change than the GrIS.

AIS ice mass balance is determined by ice discharge (D) and surface mass balance (SMB). The latter includes precipitation, sublimation and meltwater runoff, but sublimation and runoff are negligible for the AIS^4^. The empirical projection of AIS mass balance and its contribution to GMSL is based on the assumption that long-term variations of AIS mass is mostly controlled by D which undergoes slow (decadal and longer) variability because significant long-term variations have not been seen in AIS precipitation *rates* during the last a few decades^3^. However, the previous study^5^ based on comparison of GRACE gravity data and a SMB reanalysis model estimated that acceleration of ice mass loss in the Amundsen Sea Embayment portion of the AIS during 2003–2013 was − 13.6 Gton/year^2^, and that precipitation decrease during the same period explained about 60% of this (− 8.2 Gton/year^2^). Precipitation effects on AIS mass balance (i.e. SMB) should be carefully considered in order to understand multi-decadal and longer AIS mass changes, given that precipitation tends to vary on relatively long climate oscillation time scales. Therefore, abrupt AIS mass loss during the last decade found in IMBIE2 needs to be understood by separating contributions of D and SMB.

The objective of this study is to examine AIS SMB since 1979 using the state-of-the-art numerical model and its contribution to AIS mass balance. We show that inter-annual and longer SMB variations are significant. We also find a bi-polar SMB pattern associated with a precipitation decrease in the Pacific sector, and increases in Atlantic and Indian sectors, which is highly correlated with the Southern Annular Mode (SAM). AIS SMB variations produce apparent abrupt AIS mass loss, and after correcting for the SMB contribution, AIS mass loss associated with D shows a steady increase.

## AIS SMB from 1979 to 2017

Precipitation *rates* (1979–2017) of numerical models show common variation in sub-annual and annual time scales (see “Methods” and Supplementary Fig. S1). This may be partly because that they are commonly forced by the European Centre for Medium Range Weather Forecasts (ECMWF) reanalyses^6,7^. There are minor inter-annual and longer period variations, and long-term trends are not statistically significant^8^. The linear trend of precipitation *rates* from ECMWF Reanalysis 5th Generation (ERA5)^9^ is estimated to − 0.1 ± 0.5 Gton/year with a 95% confidence interval, and other models provide similar near-zero rates. As a result, it has been thought that the role of precipitation in long-term AIS mass variation can be ignored^3^. We use the most recent state-of-the-art reanalysis, ERA5, to investigate this.

**Figure 1:**
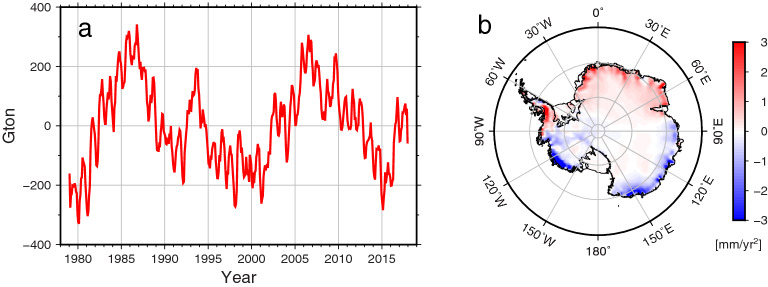
(**a**) Antarctic accumulated precipitation after removing a linear trend (1979–2017). The linear trend is estimated from the first order polynomial (${a}_{0}+{a}_{1}t$) fitting to accumulated precipitation. (**b**) Map of acceleration rate of accumulated precipitation. Acceleration rates are estimated from the second order polynomial (${a}_{0}+{a}_{1}t+\frac{1}{2}{a}_{2}{t}^{2}$) fitting to accumulated precipitation. Figures are created with Generic Mapping Tools (GMT-5.4.5, https://gmt.soest.hawaii.edu/).

AIS mass change (M) is approximately determined by ice discharge (D) and SMB, related by $M=SMB-D$. Precipitation *accumulation* (time integration of precipitation) contributes to AIS mass change in SMB. Small long-term precipitation variations will tend to be amplified, while shorter period but larger sub-annual and annual fluctuations will tend to be suppressed by integration. A small negative linear trend in precipitation rate, for example, can produce a significant acceleration in ice mass loss. Figure 1a shows accumulation of precipitation (approximately equivalent to SMB). A linear trend was removed from accumulated precipitation time series, equivalent to removing the mean value in precipitation rate, which is not the focus of this study. Detrended SMB ($\Delta$ SMB) from ERA5 shows inter-annual and longer variations^10^, but an acceleration rate in $\Delta$ SMB is minor over the entire study period (1979–2017). We estimated acceleration at grid points to obtain acceleration maps in Fig. 1b. Only statistically significant values (non-zero within a 95% confidence interval) are plotted. Acceleration rates in most regions are significant despite their small spatial average over the entire AIS (− 0.3 Gton/year^2^). For example, a positive acceleration of about 2 Gton/year^2^ is shown in Atlantic and Indian Sectors while a negative acceleration of about − 2 Gton/year^2^ is found in the Pacific Sector. A spatial pattern of significant acceleration bisects the entire AIS.

AIS SMB shows significant variability at inter-annual to decadal time-scales as indicated in Fig. 1. Because such variability is important to ongoing AIS ice mass change, it is important to understand its underlying causes. Medley and Thomas^11^ reviewed the two causes of long-term variations in AIS precipitation rates, thermodynamic processes and atmospheric circulation. The first is associated with a near exponential increase in water vapor with increasing air temperature (Clausius-Clapeyron relation) and would induce a long-term positive trend in precipitation rate over the past 100 years^12^. However, AIS $\Delta$ SMB acceleration (− 0.3 ± 0.2 Gton/year^2^) is small (implying a near-zero trend in precipitation rate). This indicates relatively small thermodynamic process contributions to AIS precipitation during this period (1979–2017).

**Figure 2:**
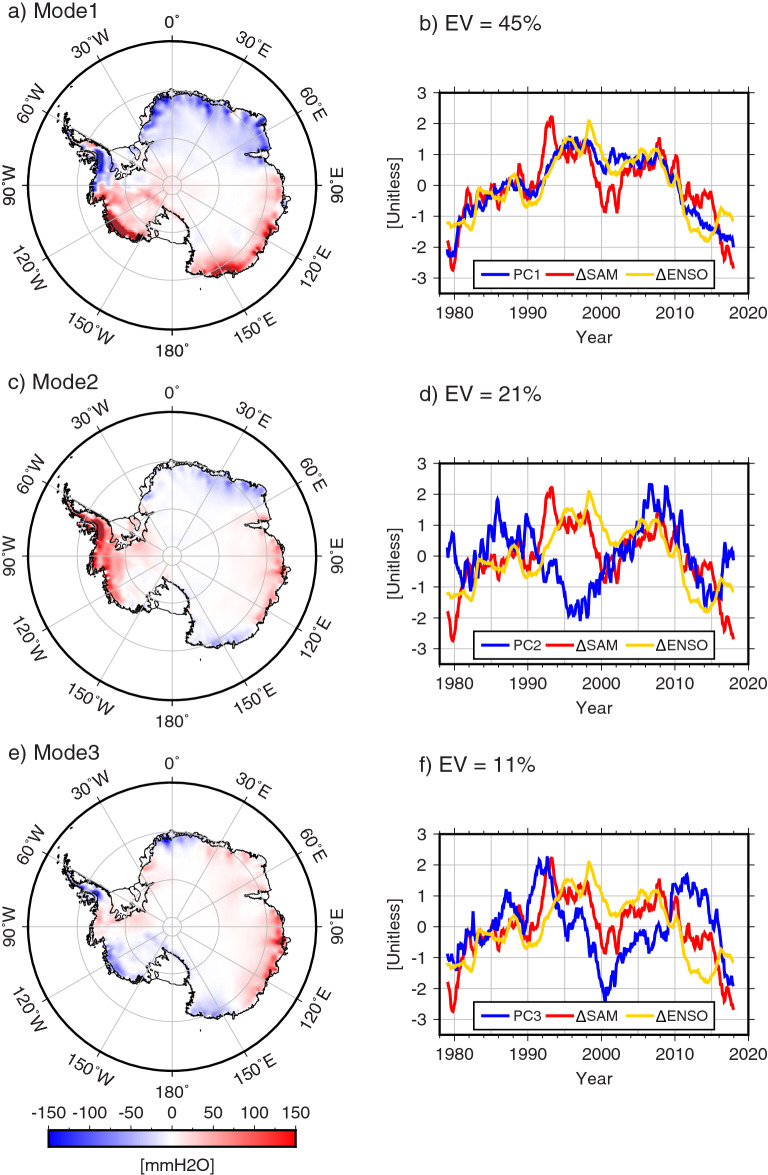
Leading three modes of EOF analysis from AIS $\Delta$ SMB (accumulated precipitation). Each of red and yellow lines in right panels show $\Delta$ SAM (time-integrated) and $\Delta$ ENSO (time-integrated), respectively. For clarity, both $\Delta$ SAM and $\Delta$ ENSO are displayed reversed in sign. Figures are created with Generic Mapping Tools (GMT-5.4.5, https://gmt.soest.hawaii.edu/).

SAM and El Ninõ-Southern Oscillation (ENSO), are primary modes of atmospheric circulation variability in Southern high latitudes^13,14^. For example, SAM modulates low pressures around Antarctica and thus alternates warm/moist and cold/dry air advection^14^. As a result, the previous study^11^ suggested that a positive trend in SAM is a cause of a decrease in AIS net precipitation rate. To examine this, we first use ordinary EOF analysis on the area-weighted $\Delta$ SMB field determined from ERA5 model (see “Methods”). Figure 2 shows the three leading EOF modes explaining 77% of $\Delta$ SMB variance. The left panels are the spatial patterns of each mode. Blue lines in the right panels show corresponding PCs. Time-integrated SAM and ENSO indices can also be compared with $\Delta$ SMB (time integration of precipitation rate). Time-integrated ENSO indices have also been used to examine ENSO effects on ice shelf volume changes^15^. Red and yellow lines in the right panels show time-integrated SAM and ENSO indices after removing linear trends ($\Delta$ SAM and $\Delta$ ENSO, respectively) as done for $\Delta$ SMB. The first mode PC shows a long-term variation similar to $\Delta$ SAM and $\Delta$ ENSO (Fig. 2b) confirming that Antarctic precipitation is affected by SAM and ENSO^13,14^. However, there is no apparent inter-annual variability in the first PC unlike both indices. On the other hand, second and third mode PCs partly show similar inter-annual variations as $\Delta$ SAM during the latter (2010–2015) and former (1979–2000) periods, respectively (Fig. 2d,f), suggesting that $\Delta$ SMB signals modulated by $\Delta$ SAM are mapped into multiple EOF modes. It is difficult to interpret the multiple modes if this is the case.

**Figure 3:**
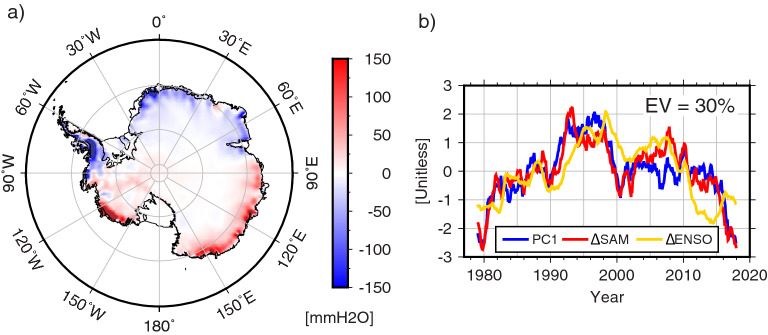
Spatial (**a**) and temporal (**b**) patterns of the first REOF mode calculated from the leading three EOF modes of AIS $\Delta$ SMB. Red and yellow lines in (**b**) shows $\Delta$ SAM and $\Delta$ ENSO, respectively. For clarity, $\Delta$ SAM and $\Delta$ ENSO are displayed with reversed sign. Figures are created with Generic Mapping Tools (GMT-5.4.5, https://gmt.soest.hawaii.edu/).

We can apply rotated EOF (REOF) analysis to map ordinary EOF modes into other modes by rotating associated basis functions (see “Methods”). If AIS $\Delta$ SMB is dominated by $\Delta$ SAM or $\Delta$ ENSO (i.e., precipitation rate is dominantly affected by SAM or ENSO^14^), the first REOF mode PC should be similar to $\Delta$ SAM or $\Delta$ ENSO. The left panel of Fig. 3 shows the first REOF spatial mode. The blue line in the right panel is its temporal variation, and the red and yellow lines show $\Delta$ SAM and $\Delta$ ENSO, respectively. The explained variance of the first REOF mode is 30%. The first REOF mode spatial pattern is similar to that of the first EOF mode but the first mode REOF PC shows a very similar variation to $\Delta$ SAM (with a correlation coefficient of 0.89). $\Delta$ ENSO also shows similar variations to the REOF PC but their correlation (0.62) is lower than for $\Delta$ SAM, which is consistent with the previous study^14^. These results show that since 1979 SMB has been mainly modulated by SAM, producing a bi-polar pattern: acceleration of ice mass loss in the Pacific Sector and acceleration of gain in Atlantic and Indian Sectors. Similar result can also be obtained by regression analysis (see “Methods”). $\Delta$ SMB modulated by $\Delta$ SAM can be predicted by a linear regression between the two at each grid point during the study period. Figure S2 shows the resulting prediction which is very close to the spatial pattern and amplitude shown in Fig. 3a.

**Figure 4:**
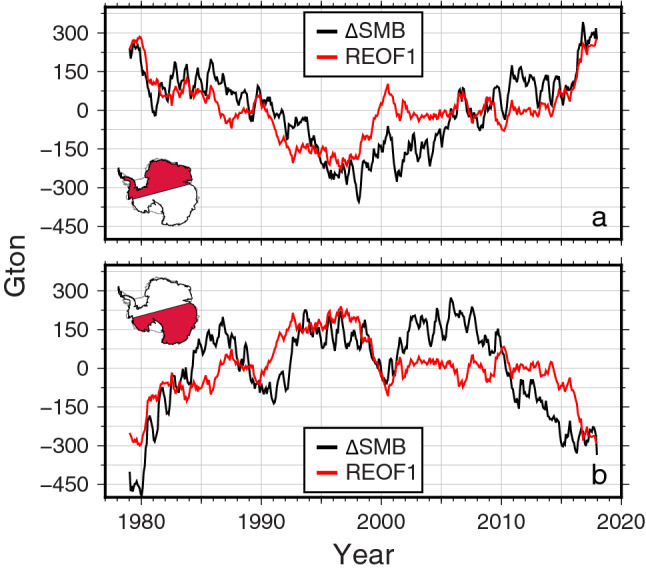
Comparison of $\Delta$ SMB (black) and reconstructed $\Delta$ SMB (red) for the first REOF mode over the Pacific Sector (**a**) and Atlantic-Indian Sector (**b**). Figures are created with Generic Mapping Tools (GMT-5.4.5, https://gmt.soest.hawaii.edu/).

Figure 4 shows total $\Delta$ SMB (black) from ERA5 and reconstructed $\Delta$ SMB modulated by SAM from the first REOF mode (red) over the two AIS regions considering the bi-polar pattern in Fig. 3a. Black ($\Delta$ SMB) and red (reconstructed $\Delta$ SMB from REOF1) show similar multi-decadal variations in both regions. The acceleration rate of $\Delta$ SMB shown in the top panel is 2.1 ± 0.1Gton/year^2^, and that of $\Delta$ SMB affected by SAM is 1.5 ± 0.1 Gton/year^2^, which explains 72% of the multi-decadal variability. Similarly, in the region with the negative acceleration (bottom panel), 67% of the acceleration (− 1.6 ± 0.1 Gton/year^2^ out of − 2.4 ± 0.2 Gton/year^2^) is explained by SAM. This shows that there are large inter-annual and longer SMB variations over AIS, and among them, multi-decadal variations are affected by SAM.

## Observation of AIS SMB

ERA5 model predictions of SMB variations can be validated using space and in-situ observations. ERA5 SMB predictions for 1979–2000 show pronounced multi-decadal ice mass loss acceleration of − 5.7 ± 0.9 Gton/year^2^ (Fig. 1) for the entire AIS. Similar evidence has been found in the ice core records^11^. A precipitation decrease since 1979 has been found in dozens of ice-cores collected throughout Antarctica^16^; AIS SMB acceleration due to the 1979–2000 precipitation decrease was − 2.7 ± 3.8 Gton/year^2^.

**Figure 5:**
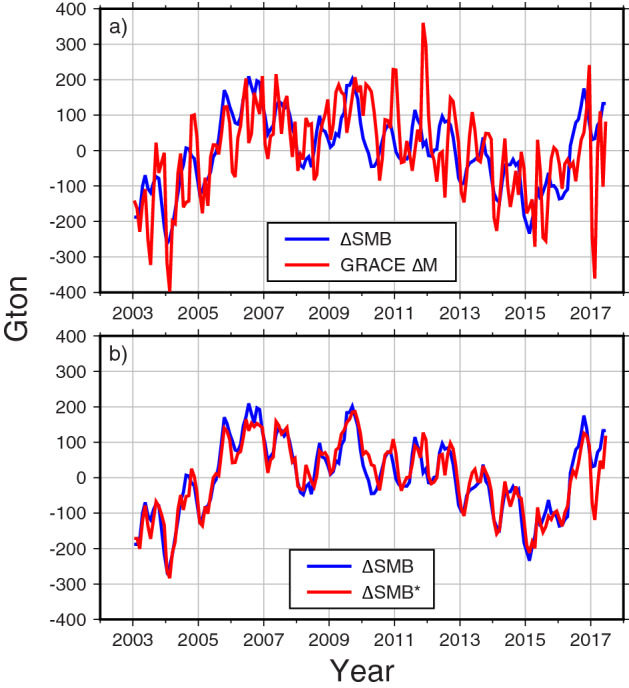
(**a**) Comparison between $\Delta$ SMB (blue) and GRACE mass estimates $\Delta$ M (red). (**b**) Similar to (**a**) but $\Delta$ SMB^*^ is the estimated $\Delta$ SMB from $\Delta$ M after ice discharge and barometric pressure error corrections. Figures are created with Generic Mapping Tools (GMT-5.4.5, https://gmt.soest.hawaii.edu/).

Satellite gravimetry (GRACE) observations of monthly surface mass change from April 2002 to June 2017, can be directly compared with model-based AIS SMB estimates. The previous study^5^ compared GRACE data with ERA-Interim SMB over the period 2003–2013. Here we undertake a similar comparison, now using ERA5 from January 2003 to June 2017, together with CSR mascon solutions^17^. Similar to Fig. 1a, we removed linear trends from GRACE AIS mass change ($\Delta$ M), with the resulting time series in Fig. 5a. $\Delta$ M shows higher frequency variability than $\Delta$ SMB, due to GRACE noise and possibly errors in atmospheric pressure corrections^5^. The acceleration rate in $\Delta$ M (− 8.6 ± 2.1 Gton/year^2^) is also higher than that of $\Delta$ SMB (− 5.1 ± 1.9 Gton/year^2^). This is likely because $\Delta$ M includes both $\Delta$ D (D after linear trend removal) and $\Delta$ SMB. Satellite remote sensing has shown that acceleration due to increasing D throughout Antarctica is about − 7.0 Gton/year^2^^18^. After subtracting $\Delta$ D from $\Delta$ M, we obtain a GRACE value for $\Delta$ SMB. This observation ($\Delta$ M − $\Delta$ D) is about − 1.6 Gton/year^2^, much smaller than the ERA5 estimate of $\Delta$ SMB. The disagreement is possibly due to atmospheric pressure error. In an earlier GRACE solution (CSR RL05), there was an apparent ice mass *positive* acceleration^5,19^ associated with mis-modeled barometric pressure over AIS. Because the synoptic scale of barometric pressure is distinct from relatively smaller spatial scale of ice discharge and SMB, the pressure error can be estimated by Empirical Orthogonal Function (EOF) analysis (see “Methods”). CSR mascon solutions also suffer from apparent ice mass positive acceleration due to barometric pressure errors, as in previous GRACE data.

We estimate $\Delta$ SMB based on GRACE observations ($\Delta$ SMB^*^) after subtracting discharge acceleration rate and correcting for barometric pressure error. This $\Delta$ SMB^*^ (from GRACE) and the ERA5 estimate of $\Delta$ SMB are shown in Fig. 5b. They agree well at inter-annual and longer periods, confirming the problem with barometric pressure in GRACE estimates, and more importantly indicating that $\Delta$ SMB prediction from numerical models reasonably depicts AIS SMB variations.

## Implications of SMB to present-day ice mass loss in AIS

Recent efforts to understand AIS ice mass change and effects on sea level rise are largely based on satellite geodetic observations^18,20,21^. We can use those observations to examine AIS mass loss associated with ice dynamics, which reflects processes such as ocean circulation, basal melting and grounding line migration^22,23^. Ice-dynamic variation time scales are likely much longer than those of SMB, and thus the current state is important in projecting future AIS mass loss and resulting sea level rise. Ice discharge (D), as a measure of ice dynamics, can be estimated from the difference between ice mass change and SMB.

**Figure 6:**
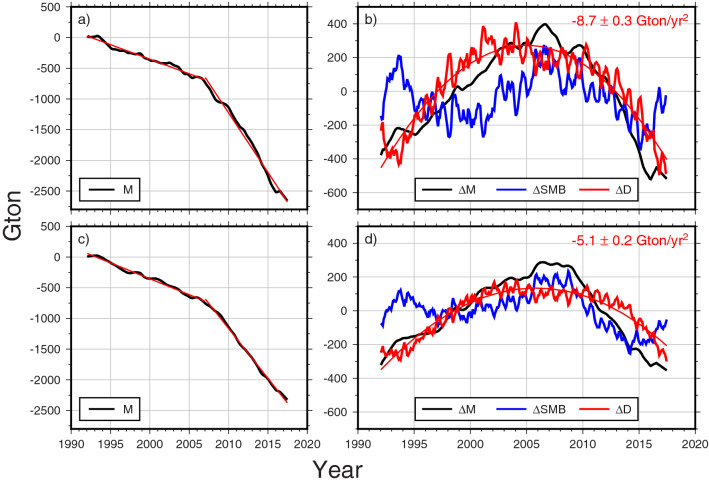
(**a**) AIS ice mass change from IMBIE2 estimates (black). Red lines represent linear trend fits before and after 2007. (**b**) Detrended variability of ice mass $\Delta$ M (black), $\Delta$ SMB (blue) and $\Delta$ D (red). The red number indicates the acceleration rate estimated from the second order polynomial (${a}_{0}+{a}_{1}t+\frac{1}{2}{a}_{2}{t}^{2}$) fitting to $\Delta$ D. (**c**,**d**) are similar to (**a**,**b**) except for West Antarctica. Figures are created with Generic Mapping Tools (GMT-5.4.5, https://gmt.soest.hawaii.edu/).

A comprehensive AIS mass change estimate using multiple satellite geodetic observations and varied processing schemes was examined in IMBIE2^3^. The black line in Fig. 6a shows that IMBIE2 AIS mass (M) loss has abruptly increased since 2007; negative trends are − 47 ± 1 Gton/year during 1992–2006 and − 194 ± 4 Gton/year during 2007–2017. The detrended IMBIE2 AIS mass change ($\Delta$ M) in Fig. 6b (black line) can be compared with ERA5 $\Delta$ SMB variations (blue line). Both $\Delta$ M and $\Delta$ SMB are obtained after removing linear trends in M (from IMBIE2) and SMB (from ERA5) during 1992–2017, respectively. The long-term $\Delta$ SMB variation (blue line) is large enough to be comparable to the detrended IMBIE2 AIS mass change ($\Delta$ M). Subtracting the $\Delta$ SMB (blue) from $\Delta$ M (black), we estimate AIS mass change associated with ice discharge, $\Delta$ D (red), with the assumption that the contribution of inland meltwater to $\Delta$ M is negligible^5^. A thin red line shows a parabolic fit to $\Delta$ D corresponding to an acceleration of − 8.7 ± 0.3 Gton/year^2^. This estimated acceleration rate with small confidence interval indicates that since 1992, AIS ice discharge has accelerated at a steady rate, rather than abruptly. The sense of the sign is that ice discharge increases every year about 8.7 Gton. We conclude that the abrupt ice mass loss in 2007 (black line in Fig. 6a) is a combined effect of $\Delta$ D and $\Delta$ SMB. Over the period 1993 to 2006, SMB mitigates ice mass loss acceleration due to D, and after that, SMB adds to acceleration in D, leading to an apparent abrupt increase in 2007. A similar interpretation can be found in ERA5 annual precipitation rate in Antarctica showing an increasing trend from 1994 to 2005 and a decreasing trend from 2006 to 2014 (Fig. S4). The timing of trend change in precipitation rate (in 2005) is different from that of $\Delta$ SMB (in 2007) because $\Delta$ SMB is the time-integration of precipitation rate.

IMBIE2 reported that the trend in Antarctic ice mass loss during 2007–2017 has abruptly increased by ~ 147 Gton/year (which is equivalent to 0.41 mm/year in global sea level change) compared to that during 1992–2006 (i.e., from − 47 Gton/year during 1992–2006 to − 194 Gton/year during 2007–2017). In this study, we find that ~ 39 Gton/year (about 27%, or global sea level change of 0.11 mm/year) out of the 147 Gton/year is attributed to SMB variation. Such a SMB effect is more significant in the West AIS, showing about 41% of the increase of ice mass loss between the two epochs (Fig. 6c,d).

## Conclusion

We examined long-term AIS SMB (accumulated precipitation) variations for 1979–2017 using precipitation fields from the ERA5 reanalysis. Even though AIS precipitation rates do not exhibit a significant trend, SMB shows strong inter-annual and multi-decadal variations. We found that multi-decadal SMB variations are related to SAM. AIS SMB modulated by SAM shows a distinct bi-polar pattern with negative acceleration in the Pacific Sector and positive acceleration in Atlantic and Indian Sectors. Model predictions of SMB variations are observed by satellite geodetic observation like GRACE. After correcting for SMB, we found a steady acceleration of ice discharge of − 8.7 ± 0.3 Gton/year^2^ for the period 1992–2017. The apparent abrupt change in 2007 is not associated with a change in ice dynamics but instead with SMB variations mostly in the West AIS.

## Methods

### Precipitation models

To examine AIS SMB, we used ERA5^9^ precipitation, an improved version of the previous ERA-Interim^7^ reanalysis. ERA-Interim has been known to represent well both sub-annual^24^ and long-term variability of AIS precipitation^8^. The horizontal resolution of ERA5 is notably improved (31 km compared to ERA-Interim, 80 km), similar level to existing regional climate models, RACMO (= 27 km)^4^ or MAR (= 35 km)^6^. We considered AIS precipitation over Antarctic drainage basins determined by satellite altimeter observations^25^. Supplementary Fig. S1 shows changes in precipitation rates (1979–2017) in Antarctica estimated from ERA5 and other models (ERA-Interim, RACMO2.3p2 and MARv3.6.4). ERA5 fluctuations in precipitation rates are similar to other numerical models^4,6,7^.

### EOF analysis of SMB

$\Delta$ SMB data are contained in a matrix of dimension of $p\times n$ (where $p$ and $n$ are the lengths of discrete spatial and temporal components, respectively). The matrix is separated into several orthogonal modes using singular value decomposition (SVD):

1 $${\Delta SMB}={\text{USV}}^{T}$$

in which U and V are orthogonal matrix with dimension of $p\times p$ and $n\times n$, respectively. The superscript T means matrix transpose. The $i$ th column of U represents the spatial pattern of the $i$ th mode, and similarly the $i$ th column of V represents the temporal variation (principal component) of the $i$ th mode. S is a rectangular matrix with only diagonal components, and each component is a singular value of each mode. If the $i$ th diagonal component of $S$ is ${d}_{i}$, the explained variance (EV) of the corresponding mode is:

2 $$EV=\frac{{d}_{i}^{2}}{\sum_{j=1}^{m}{d}_{j}^{2}}\times 100 (\%)$$

where $m$ is equal to $p$ or $n$, whichever is smaller.

### REOF analysis of SMB

Rotated EOF (REOF) analysis is a variant of EOF analysis. Typically, it may be difficult to interpret individual EOF modes because they are spatially coupled so a specific variation is contained in multiple modes. REOF transforms EOF modes into coordinate axes rotated relative to the original. To obtain rotated modes (or axes) from the original EOF modes we use a rotation matrix:

3 $$\text{W}={\text{U}}_{m}\text{R}$$

where ${\text{U}}_{m}$ is a $p\times m$ matrix consisting of *m* column vectors representing selected EOF modes. R is a $m\times m$ orthonormal matrix to rotate the EOF modes (${\text{U}}_{m}$) to new modes ($\text{W}$) while keeping their orthogonality during rotation.

The rotation matrix R needs to be determined to obtain $\text{W}$ in which vector columns are uncoupled from one another. The degree of 'uncoupling' for the $\text{W}$ can be evaluated at a geographic point with a maximum value in a single mode while values at the same point are near 0 in other modes. There are various criteria to solve such an optimization problem. Here we adopt the VARIMAX approach, the most widely used^26,27^. VARIMAX appraises the degree of uncoupling for a rotated mode based on maximizing an objective function:

4 $$f\left({W}_{k}\right)=\frac{1}{p}\sum_{i=1}^{p}{l}_{i,k}^{4}-\frac{1}{{p}^{2}}{\left[\sum_{i=1}^{p}\left({l}_{i,k}^{2}\right)\right]}^{2}$$

where ${l}_{i,k}$ is the value at *i*th grid-point explained only by the $k$ th column of $\text{W}$, i.e., ${W}_{k}$.

Practically, the $\text{W}$ (and simultaneously R) is determined using an iterative scheme. First, a column vector is calculated from the sum over each row of U_m_ and then normalized by its vector norm. This is equivalent to $\text{W}$ in Eq. (3) when using the initial R, R_0_: the first column in R_0_ is an $m\times 1$ vector of ones divided by its norm, namely ${\stackrel{\sim }{R}}_{1}$, and the other columns are all zeros. One seeks a local maximum of the objective function (Eq. (4)) by finely tuning ${\stackrel{\sim }{R}}_{1}$ iteratively. After determined the optimal ${\stackrel{\sim }{R}}_{1}$, the second and following columns (${\stackrel{\sim }{R}}_{k}$ with $k=2,\cdots ,m$) are determined in the same manner, but constrained to be orthogonal to all previous ${\stackrel{\sim }{R}}_{n}$ ($n=1,\ldots ,k-1$). Finally, rotated modes W are calculated from Eq. (3) with the optimal rotation matrix, R where $\text{R}=[{\stackrel{\sim }{R}}_{1} {\stackrel{\sim }{R}}_{2} \cdots {\stackrel{\sim }{R}}_{m}]$.

### Regression analysis between ${\Delta }$ SMB and ${\Delta }$ SAM

Let temporal variation of $\Delta$ SMB at each grid point $(x,y)$ be $B\left(x,y,t\right)$. We project normalized $\Delta SAM\left(t\right)$ (red line in Fig. 3b) onto $B$ using least-squares method. The cost-function ($J$) to estimate projected amplitude, ${a}_{1}$, is

5 $$J\left(x,y\right)={\sum_{t}[B\left(x,y,t\right)-{a}_{1}(x,y)*\Delta SAM\left(t\right)-{a}_{0}(x,y)]}^{2}$$

The amplitude of ${a}_{1}(x,y)$ is shown in Fig. S2.

### Correction of barometric pressure error from GRACE AIS mass change

For the correction of the barometric pressure error in $\Delta$ M, we apply EOF analysis to the difference between $\Delta$ M and $\Delta$ SMB, as in the previous study^5^. Before EOF analysis, both products are smoothed by a 600 km Gaussian spatial filter to suppress artifacts due to differences in spatial resolution.

Figure S3 shows spatial patterns and their principal components (PC) of the three leading EOF modes. As in the previous study^5^, mass loss acceleration signals appear over West Antarctica and the Antarctic Peninsula in the second mode. These are associated with $\Delta$ D. In the third mode, a similar signal associated with $\Delta$ D is observed around Totten glacier^21,28^. Larger spatial patterns in the second and third modes compared to major glacier outlets in AIS are due to the 600 km Gaussian smoothing. On the other hand, the first mode shows a spatial pattern with a single sign throughout Antarctica. Barometric pressure is known to have such a continent-wide synoptic spatial scale. We conclude that the continental scale first mode is likely associated with errors in barometric pressure. The first mode is subtracted from $\Delta$ M to correct this error.

## Supplementary information

Supplementary Information.

## Data Availability

ERA5 data can be downloaded from the following website: https://cds.climate.copernicus.eu/#!/search?text=ERA5&type=dataset. We used Center for Space Research (CSR) RL06 Mascon Solutions for GRACE gravity data (https://www2.csr.utexas.edu/grace/RL06_mascons.html). Monthly SAM indices are available at https://www.cpc.ncep.noaa.gov/products/precip/CWlink/daily_ao_index/aao/monthly.aao.index.b79.current.ascii. We used the Southern Oscillation Indices (SOI) as the ENSO indices, which are available at https://www.ncdc.noaa.gov/teleconnections/enso/indicators/soi/. The estimates of ice mass balance generated by IMBIE2 are available at https:// https://imbie.org/data-downloads/. RACMO2.3 can be obtained from personal contact with the Institute for Marine and Atmospheric Research (IMAU) team. Each of ERA-Interim and MARv3.6.4 is available at the following website (https://apps.ecmwf.int/datasets/data/interim-full-mnth/levtype=sfc/ , https://zenodo.org/record/2547638#.XHzH3y2B3Se), respectively.
