# Supplementary material for: Antarctic ice mass variations from 1979 to 2017 driven by anomalous precipitation accumulation
Source: Sci Rep. 2020 Nov 23;10:20366. doi: 10.1038/s41598-020-77403-5 (PMC7683593; doi:10.1038/s41598-020-77403-5)
Supplement: Supplementary file 1 — Supplementary Information. [file 41598_2020_77403_MOESM1_ESM.docx]

**Antarctic ice mass variations from 1979 to 2017 driven by anomalous precipitation accumulation**

Byeong-Hoon Kim^1,2^, Ki-Weon Seo^2*^, Jooyoung Eom^3^, Jianli Chen^4^ and Clark R. Wilson^4,5^

^1^Division of Glacial Environment Research, Korea Polar Research Institute, Incheon, 21190, Republic of Korea

^2^Department of Earth Science Education, Seoul National University, Seoul, 08826, Republic of Korea

^3^Department of Earth Science Education, Kyungpook National University, Daegu, 41556, Republic of Korea

^4^Center for Space Research, University of Texas at Austin, Austin, Texas, 78759, USA

^5^Department of Geological Sciences, Jackson School of Geosciences, University of Texas at Austin, Austin, Texas, 78712, USA

*Corresponding author. Email: seokiweon@snu.ac.kr

**Supplementary Information**

**Figure S1**. Variations of monthly precipitation rates (1979-2017) in Antarctica: ERA5 (black), ERA-interim (blue), RACMO2.3p2 (red) and MARv3.6.4 (green). Linear trends (1979-2017) of each model are shown in parentheses. Vertical offsets are applied to distinguish each time series. This figure is created with Generic Mapping Tools (GMT-5.4.5, <http://gmt.soest.hawaii.edu/>).

**Figure S2.** Prediction of $\Delta$SMB modulated by $\Delta$SAM using regression analysis. This map is created with Generic Mapping Tools (GMT-5.4.5, <http://gmt.soest.hawaii.edu/>).

**Figure S3.** Spatial patterns (a, c, e) and their PCs (b, d, f) of leading three EOF modes calculated from $\Delta$M-$\Delta$SMB. Figure are created with Generic Mapping Tools (GMT-5.4.5, <http://gmt.soest.hawaii.edu/>).

**Figure S4.** Changes in Antarctic annual mean precipitation (1979-2017) from ERA5 reanalysis. This figure is created with Matlab R2018b (<https://kr.mathworks.com/help/matlab/release-notes-R2018b.html>).
